# Disentangling informing participants from obtaining their consent

**DOI:** 10.1002/lrh2.70014

**Published:** 2025-04-21

**Authors:** Patricia Pearl O'Rourke, Joseph Ali, Judith Carrithers, David Magnus, Benjamin S. Wilfond, Sheana Bull, Laura M. Dember, Gail D'Onofrio, Julie Goldman, P. Michael Ho, Edward R. Melnick, Karen L. Staman, James A. Tulsky, Miguel A. Vazquez, Angelo Volandes, David Wendler

**Affiliations:** ^1^ Harvard Medical School, Harvard University Boston Massachusetts USA; ^2^ Berman Institute of Bioethics, Johns Hopkins Baltimore Maryland USA; ^3^ Bloomberg School of Public Health, Johns Hopkins Baltimore Maryland USA; ^4^ Advarra Inc Columbia Maryland USA; ^5^ Stanford Center for Biomedical Ethics Stanford University Stanford California USA; ^6^ Treuman Katz Center for Pediatric Bioethics and Palliative Care, Children's Hosptial, University of Washington Seattle Washington, DC USA; ^7^ Seattle Children's Research Institute, Children’s Hospital, University of Washington Seattle Washington, DC USA; ^8^ University of Washington School of Medicine, University of Washington Seattle Washington, DC USA; ^9^ Community and Behavioral Health, Health Impact Laboratory Colorado School of Public Health Aurora Colorado USA; ^10^ Renal‐Electrolyte and Hypertension Division, Department of Medicine, Hospital of University of Pennsylvania Philadelphia Pennsylvania USA; ^11^ Department of Biostatistics, Epidemiology and Informatics, University of Pennsylvania Philadelphia Pennsylvania USA; ^12^ Center for Clinical Epidemiology and Biostatistics; Perelman School of Medicine University of Pennsylvania Philadelphia Pennsylvania USA; ^13^ Emergency Medicine and Medicine Yale School of Medicine New Haven Connecticut USA; ^14^ Yale School of Public Health, Yale University New Haven Connecticut USA; ^15^ Department of Psychosocial Oncology and Palliative Care Dana‐Farber Cancer Institute Boston Massachusetts USA; ^16^ Division of Cardiology University of Colorado Anschutz Medical Campus Aurora Colorado USA; ^17^ Cardiology Section Veterans Affairs Eastern Colorado Health Care System Aurora Colorado USA; ^18^ Department of Emergency Medicine Yale School of Medicine New Haven Connecticut USA; ^19^ Department of Biostatistics (Health Informatics) Yale School of Public Health New Haven Connecticut USA; ^20^ Duke Clinical Research Institute Durham North Carolina USA; ^21^ Harvard Medical School and Department of Psychosocial Oncology Dana‐Farber Cancer Institute Boston Massachusetts USA; ^22^ Department of Medicine Brigham and Women's Hospital Boston Massachusetts USA; ^23^ Division of Nephrology University of Texas Southwestern Medical Center Dallas Texas USA; ^24^ Department of Medicine Massachusetts General Hospital Boston Massachusetts USA; ^25^ ACP Decisions Boston Massachusetts USA; ^26^ Department of Bioethics National Institutes of Health Bethesda Maryland USA

**Keywords:** bioethics, institutional review board, learning health system, pragmatic clinical trial, protection of human subjects, regulations, waiver of informed consent

## Abstract

**Introduction:**

Pragmatic clinical trials conducted in the context of routine care frequently satisfy the regulatory criteria for a waiver of research consent. When they do, investigators and Institutional Review Boards might assume that there is no reason to communicate any information regarding the study to participants. Yet, this approach ignores the possibility that there may be value in providing information to participants, even when the study does not pose significant risks and researchers are not obtaining their consent.

**Methods:**

Members of the NIH Collaboratory Ethics and Regulatory Core working group used ethical analysis to determine whether there are reasons to provide information to research participants, other than notifying them of significant risks or obtaining their consent. Study team members then provided examples of trials which illustrate the feasibility and different options for providing information to participants in the context of trials conducted with a waiver of research consent.

**Results:**

Communicating information to participants can promote one or more of six goals: respect for persons, participant understanding of the research, participant understanding of their contributions, participant ability to voice any concerns, participant engagement, and trust and trustworthiness. Providing information can also raise potential concerns about feasibility and cost, which need to be balanced against these reasons to inform participants. Depending on the study, a variety of methods can be used to communicate information; for example, letters, email, flyers, posters, as well as brief conversations with clinicians.

**Conclusion:**

Even when researchers are not obtaining participants' consent, communicating information can promote one or more of six important goals. Providing information to participants should thus be the default for trials conducted under a waiver of research consent.

## 1 INTRODUCTION

Pragmatic clinical trials (PCTs) are designed to assess the comparative risks and benefits of biomedical or behavioral health interventions in real‐world settings, with the goal of informing decision makers.^1^ PCTs that are conducted in the context of routine care frequently satisfy the regulatory criteria for a waiver of research informed consent.^2^ When they do, and a waiver is approved, investigators and reviewers might assume that there is no reason to communicate participants any information about the study to participants. This approach might be defended on the grounds that waivers of research consent are permitted only when a study poses very low risks. Informing participants of significant risks, one of the primary functions of research consent, is thus less important or does not apply at all.

However, this approach ignores the possibility that providing participants with information can be valuable independent of describing significant risks or obtaining their consent. For example, the Ottawa Statement on the Ethical Design and Conduct of Cluster Randomized Trials argues that providing information about a study which qualifies for a waiver of research consent offers a way to demonstrate respect for persons.^3^ The *Council for International Organizations of Medical Sciences* (CIOMS) guidelines specify that it is important to “preserve as much of the informed consent process as possible” when consent is waived.^4^ Despite these endorsements, very little has been written on whether and why researchers should provide participants with information about studies which are conducted with a waiver of research consent.

## 2 QUESTIONS OF RESEARCH INTEREST

We assessed whether, why, and how researchers should notify participants about studies that are conducted under a waiver of research consent.

## 3 METHODS

The NIH Pragmatic Trials Collaboratory^5^ supports the design and conduct of 32 large‐scale PCTs involving more than 1400 sites, 1.2 million participants, and 14 NIH Institutes and Centers across 49 states. As part of the Ethics and Regulatory Core Working Group of the Collaboratory, our mission is to explore areas of ethical uncertainty and develop approaches that foster the ethical design and conduct of pragmatic trials. One area of uncertainty was whether there are reasons to communicate information to research participants, other than notifying them of significant risks or obtaining their consent. As part of our assessment of this issue, we consulted the investigators of Collaboratory studies that provided participants with information despite being granted a waiver of research consent in order to assess the feasibility and options for providing this information.

## 4 HUMAN SUBJECTS PROTECTIONS

The studies described in this manuscript, conducted by the NIH Collaboratory, were reviewed and approved by Institutional Review Boards.

## 5 RESULTS

Our analysis suggests that providing information to participants about studies that are conducted with a waiver of consent can promote six important goals: respect for persons, participant understanding of the research, participant understanding of their contributions, participant ability to voice any concerns, participant engagement, and trust and trustworthiness. To illustrate the feasibility of communicating with participants and the options for doing so, we describe pragmatic trials that notified participants despite being granted a waiver of the requirement to obtain research consent. Finally, we consider potential problems that can arise as a result of notifying participants.

The extent to which informing participants can promote the six goals, and the potential problems associated with doing so, depends on the study. Given this variability, it is not possible to identify specific information which should be provided about all studies, nor how it should be communicated. With this in mind, we focus on describing the six goals, the ways in which information can be provided, and the potential costs of doing so. We hope this analysis puts investigators and reviewers in a position to determine, on a case‐by‐case basis, whether and how to notify participants regarding the study in question. While we focus on studies which qualify for a waiver of research consent, the six goals are relevant to trials that obtain research consent as well.

## 6 WAIVING RESEARCH CONSENT

Informed consent is one of the primary protections for research participants. Nonetheless, most regulations permit the requirement to obtain research consent to be waived in certain circumstances. Under US regulations, for example, institutional review boards (IRBs) may waive the requirement to obtain research consent when a study satisfies five criteria (Table 1), which are frequently satisfied by PCTs.

**TABLE 1:** US regulatory criteria for waiving or altering research consent.^6^

1	The research involves no more than minimal risk to the subjects
2	The research could not practicably be carried out without the requested waiver or alteration
3	If the research involves using identifiable private information or identifiable biospecimens, the research could not practicably be carried out without using such information or biospecimens in an identifiable format
4	The waiver or alteration will not adversely affect the rights and welfare of the subjects; and
5	Whenever appropriate, the subjects or legally authorized representatives will be provided with additional pertinent information after participation

Consider a case in which there are two interventions in routine use for a given condition. Both are used widely by competent and experienced clinicians, but there are no comparative data to determine whether one is safer or more effective than the other. In this setting, PCTs conducted in the context of routine care can provide valuable data on the safety and effectiveness of the interventions as they are used in the clinical setting.

With this in mind, imagine that researchers propose a comparative trial which randomizes eligible institutions or eligible patients to one of the two interventions. The researchers propose to obtain standard clinical consent, discussing the patient's condition and, as is routine, explaining the intervention they will receive. The researchers do not plan to describe the nature or goals of the research, however, and ask the IRB to waive the requirement to obtain research consent. The reviewing IRB finds that the study satisfies the five criteria and agrees. In particular, the IRB finds that the study poses no added research risk. It also finds that the study could not “practicably” be carried out if the investigators were required to obtain research consent on the grounds that obtaining it would likely yield a biased study sample.^7^, ^8^ or because the cost and complexity of this activity are not reasonable.

Whether a study satisfies the five criteria for waiving research consent is a matter of judgment, and some may disagree with this determination. For example, there is significant debate over the extent to which randomization, by deviating from clinical judgment, poses risks to participants beyond the risks of standard care.^9^ In addition, US regulations permit IRBs to waive research consent only when doing so will not “adversely affect the rights and welfare of the subjects.” Some might argue that, in all cases, individuals have a right to decide for themselves whether to participate in research. On these grounds, or others, some might argue that it is not appropriate to waive research consent.

For present purposes, we will not consider these arguments. Our goal is not to determine when it is appropriate for IRBs to waive research consent. Instead, the goal is to consider whether and to what extent researchers should communicate information to participants when the requirement to obtain research consent has been waived. Answering this question is important because, as noted earlier, investigators and IRBs might assume that there is no reason to provide information about studies which the IRB regards as posing little to no added research risks.

The present proposal to notify participants about studies conducted with a waiver of research consent is consistent with the 5th criterion in the US regulations that studies must satisfy to be granted a waiver: “Whenever appropriate, the subjects or legally authorized representatives will be provided with additional pertinent information after participation.” This criterion refers to providing information after a study is completed, not to providing information before or during the study. This criterion is also frequently interpreted as limited to debriefing participants following deceptive studies.^10^ Still, this criterion recognizes that, when the requirement to obtain research consent is waived, there can still be reason to provide participants with some information. It thus raises the general question of *why* researchers should provide information about the study to participants when it does not pose significant risks, and the researchers are not soliciting their research consent. Specifically: beyond debriefing participants following deception studies, are there other reasons to provide participants with information about studies for which the requirement to obtain research consent has been waived? Members of the NIH Collaboratory Ethics and Regulatory Core working group used ethical analysis and the experience of researchers conducting pragmatic trials to try to answer this question.

## 7 SIX GOALS

### 7.1 Respect for persons

The Belmont Report recognizes respect for persons as one of the fundamental ethical principles for clinical research. It understands the principle in terms of two moral requirements: “the requirement to acknowledge autonomy and the requirement to protect those with diminished autonomy.”^11^ In this way, the Belmont Report essentially equates respect for persons with respect for their agency or autonomy. And that view suggests: when research consent is not being obtained, respect for persons offers no reason to provide participants with any information about the study. However, research participants are more than just autonomous agents who make decisions about the course of their lives and respecting them in the context of clinical research goes beyond obtaining their research consent.

Respect involves recognizing and taking seriously the fact that individuals have their own perspectives, experiences, preferences, values, and goals.^12^ For example, empirical studies find that many individuals want to receive information even when the requirement to obtain research consent has been waived. In particular, they want to know whether they are participating in research.^13^ This suggests that notifying participants that they are enrolled in a study provides a way to respect them and their preferences.^14^, ^15^, ^16^

Determining the implications of this broader understanding of respect for persons is complicated by a lack of consensus on the relationship between respect for persons and other ethical principles. Some philosophers regard respect for persons as the primary or even sole principle in ethics, with all other ethical principles following from it.^17^ Others, especially consequentialists, regard respect for persons as important only to the extent that it promotes one or more other goals, such as increased pleasure or greater happiness.

There is further debate over the relative weight or significance of respect for persons compared to other ethical principles or considerations. Strict libertarianism implies that respect for persons always takes precedence over other ethical considerations. In the context of clinical research, this view suggests that it is never appropriate to waive research consent, even when doing so offers the potential for significant social value at little risk to participants. In contrast, consequentialism implies that respect for persons should be ignored whenever doing so promotes better outcomes. This view suggests that it can be acceptable to waive research consent even for a study that poses very high risks, when doing so offers the opportunity to realize significant social value which cannot be otherwise obtained.

Given this lack of consensus, most research regulations do not endorse a specific theory or way of understanding respect for persons and its implications. Instead, they take a pragmatic approach, prohibiting studies that seem clearly unacceptable (e.g., pediatric studies that pose high risks and offer no chance for participant benefit) and relying on IRB judgment to determine whether or not to approve the others. For example, US regulations stipulate that IRBs may approve a waiver of research consent only when the rights and welfare of the participants will not be adversely affected.^18^ But the regulations do not specify when a waiver of research consent might adversely affect participants' rights and welfare. Instead, the regulations rely on the IRB's judgment.

We take a similar pragmatic approach here, describing the relevant goals and their implications for informing participants without appeal to a general (and inevitably controversial) moral theory which specifies how the different goals are related to one another. Specifically, we recommend that, in the context of studies conducted with a waiver of research consent, researchers and review committees should adopt a default of notifying individuals that they are involved in research. When to consider exceptions to this default should be based on the importance of the information to and for the participants, and the significance of any competing factors, especially the feasibility and costs of providing it.

### 7.2 Understand activity and relationships

Ideally, the public would be aware of and comfortable with the conduct of clinical research. They would recognize the importance of clinical research, especially the need for ongoing evaluation and study to improve medical care.^19^ They would also have some understanding of the different types of studies and how they are conducted. Unfortunately, this ideal has yet to be achieved, and we face a baseline of poor “research‐literacy.” Communicating to individuals that they are involved in a study provides an opportunity to improve awareness and understanding of clinical research.^20^, ^21^

In addition, individuals have an interest in understanding the nature of the activities and relationships in which they are engaged. This is one of the lessons of Robert Nozick's famous Experience Machine, which uses electrodes and sophisticated computers to give individuals the pleasurable illusion of having a good life—making friends and accomplishing great things—while they are actually floating in a tank in a neurology laboratory doing nothing.^22^ Nozick points out that this would be an undesirable existence because it is not valuable for us simply to have pleasant experiences. It is valuable for us to actually do things, to engage with others and the world. And it is important to understand the nature of the activities and relationships in which we are involved. In the present context, providing information to participants can help to promote this goal. It can, for example, enable participants to recognize that those with whom they are interacting and who are reviewing their health information are not just providing clinical care; they are also conducting research.

### 7.3 Understand contributions

Some might argue that individuals who are enrolled in a study without research consent are not actively participating in the study. Instead, they are being exposed to research procedures and should be described as “subject” rather than “participant” However, it is not obtaining research consent which makes individuals contributors to a study—it is enrolling them in the study and collecting data from them. We thus use the term “participants” to underscore the fact that these individuals are contributing to the research. This fact also provides a reason to inform them that they are participating in research, and to explain the goals of the study so they can understand the activity in which they are participating.

### 7.4 Voice any concerns

Obtaining research consent provides individuals with an opportunity to voice and discuss any concerns they might have with a study; waiver of research consent, without notification, eliminates this opportunity. Of course, a waiver of research consent is allowed only when a study poses no more than minimal risk, and the waiver does not adversely affect the rights and welfare of participants. Hence, one might respond that there is little chance that involvement in the study will raise concerns for any participants.

While this makes sense, IRBs determine whether the conditions for waiver are satisfied before the study begins, and they do so based on the target population in general, not the individuals who are enrolled. It follows that participation may raise concerns for specific individuals, even when the study qualifies for a waiver of research consent. Informing individuals about the research provides a means to partially reinstate the opportunity for them to voice and discuss any concerns. This process can also provide a means for researchers to learn about potential concerns that might merit a change in the study design or conduct.

Informing individuals that they are being enrolled in a study also raises the possibility that some individuals will request to not be enrolled or to opt‐out. Permitting individuals to opt‐out provides a way to at least partially respect their autonomy, even when full research consent is not being obtained.^23^, ^24^, ^25^ Of course, permitting individuals to opt‐out can also undermine the value and/or validity of a study. How to balance these issues depends on the importance for individuals of being able to decide whether to participate in the study. For example, the more the study deviates from standard care, the more reason there is to permit individuals to opt‐out.

Making this determination also depends on the importance of the study and the extent to which some opt‐outs would undermine its value or validity. As a result, it is not possible to give a general principle for when opt‐out should be permitted and when it shouldn't be permitted. Instead, this is another issue on which IRBs will have to use their judgment. At a minimum, there should be a compelling reason for not permitting individuals to opt out. Researchers and review committees should determine prospectively how to respond to individuals who ask to opt out. This should include whether opt‐out is possible and, if not, what alternatives an individual may have.

### 7.5 Promote participant engagement

Informing participants provides a means to engage them and gain their support for the study. This may be particularly important for longitudinal studies in which there is some level of participant interaction. For example, if a study includes surveys or text messages, describing the study can provide context for these activities and alert participants in advance to expect them. Similarly, if a study is evaluating the efficacy of a medication when it is taken as prescribed, offering information gives investigators the opportunity to emphasize the importance of following the prescription. For studies that assess the side effects of standard treatment, notification provides a means for investigators to emphasize the importance of accurate reporting.

Importantly, studies find that willingness to participate is greater among those who have some understanding of the research and its importance.^25^, ^26^ These data underscore the benefits of providing information and tailoring it to the specific trial. For example, instead of simply disclosing the fact that individuals are being enrolled in a study, it can be important to explain why the specific trial is valuable and who may benefit from it.

### 7.6 Trust and trustworthiness

Communicating information to participants when it is not required by the regulations provides a means to establish the trustworthiness of the researchers. It can also help to develop and/or maintain trust in research and researchers. Specifically, providing information to participants, especially if this practice is adopted generally within an institution, assures the public that research is not being conducted clandestinely within that institution. In contrast, individuals who learn that research is conducted without participants being informed or learn that they were enrolled in research without their knowledge may assume they were not informed because the research was risky or ethically problematic. They may also wonder whether they are involved in a research study at times when they are simply receiving standard care. This, in turn, may foster a lack of trust in researchers and clinicians.

## 8 IMPLEMENTATION

Current practice is often based on the view that meaningful consent requires full regulatory consent with all the required elements. And, if that is not possible, there is no reason to inform participants at all. This view ignores the fact that providing some information can be valuable even when the researchers are not obtaining participants' research consent. In particular, we have argued that providing participants with information can promote one or more of six goals.

The relevance and importance of these goals vary across studies. For example, promoting participant engagement can be critical in a study that depends on patients accurately reporting outcomes, but might be irrelevant for a study designed to assess how to increase adoption of best practices for sedated and intubated patients. As a result, rather than trying to identify certain information that should be provided by all studies, it will be important for researchers and review committees to make this determination based on the specific study in question. A number of factors influence the success of realizing these goals; two major ones are the amount of information provided and how it is presented. First, consider the amount of information.

At one end of the spectrum are general statements that research is being conducted at an institution, but with no details about specific studies; for example, placing posters in waiting rooms that state that research is sometimes conducted at the institution. This may offer a way to convey trustworthiness for research in general and enable individuals who have significant concerns to express them to institutional representatives.

For example, the ACP‐PEACE study is being conducted to increase advance care planning in the elderly using training tools (clinician communication skills training and patient video decision aids), and completion of advance care planning is the primary outcome.^27^ For notification, posters are placed in the clinical setting stating that anonymous data from the electronic health record is being used for research to improve care and that data security is provided. No study‐specific information was provided on the poster, but a phone number was provided to call for questions or to opt out. This approach informs patients that their data could be used for research.

At the other end of the spectrum are notifications that include many, if not most, of the regulatory elements of informed consent, such as the research goals, procedures, risks, potential benefits, privacy protections, and sponsors. The TiME trial was conducted to determine whether a minimum session duration of 4.25 h for maintenance hemodialysis improves outcomes compared with usual care.^28^ For notification, a written document was provided to each eligible patient in the participating dialysis facilities, and posters were placed in the facility with information about the purpose of the research, trial procedures, confidentiality protections, frequently asked questions, and contact number for any questions or to opt out. The goal of this notification was to inform patients that their facility is participating in a research study, explain the purpose of the trial, and provide an option for them to opt out of participation.

In between these two extremes are notifications that are specific to the research study but provide less information. The ICD‐Pieces trial was conducted to help primary care physicians deliver evidence‐based interventions to treat patients with coexisting chronic kidney disease, diabetes, and hypertension.^29^ For this trial, notification was provided via posters, flyers, notices, and hand‐outs with frequently asked questions that were placed in the clinic and/or given to participants. These notices contained information about the sponsor, details about the research, confidentiality protections, and a contact number for any questions or to opt out. The goal of notification was to inform patients that the practice was part of a research study and to provide patients and providers the opportunity to opt out of participation in the study or use of their data.

As another example, the Nudge study was conducted to improve adherence to chronic cardiovascular medication via text messaging.^30^ In this case, letters were sent to all participants with frequently asked questions, information about the sponsor, the purpose and details of the research, confidentiality protections, and contact numbers for any questions or to opt out. The goals for notification were to inform subjects of possible text messages in order to avoid confusion and to provide patients the opportunity to opt out of participation in the study or the research use of their data.

As a final example, physicians are sometimes the subjects of PCTs. This was the case with the EMBED trial, which was designed to support the use of buprenorphine for opioid use disorder in the emergency department via a clinical decision support tool.^31^ To alert physicians and patients about the use of the clinical support tool, clinician‐ and patient‐facing posters were placed in common areas in the emergency department. Clinicians also received blast emails and presentations at staff meetings with details about the study and a contact number for questions or to opt out.

A second factor that can have a significant influence on the promotion of the six goals is the method of communication. Focused conversation with study staff is perhaps the best way to be confident the information was understood.^19^ Individual directed communication, by letter, email, or personally delivered flyers, makes it likely that the information is received, but does not ensure that the information was read and/or understood. Input from participants can provide a valuable perspective regarding the best approach to notification.

The advantages of the different approaches must be weighed against the cost of developing and supporting them. For example, postage, personnel to deliver flyers and/or talk with individuals can be expensive. Placement of posters or flyers available for individuals to pick up (not personally delivered), while easier to implement, raises the question of how often they are seen and actually read. There are also strategies regarding where to place posters and/or flyers: most effective is placement in the specific care setting where the research is being conducted, as was done for TiME, ACP‐PEACE, and EMBED. In contrast, if placed in public areas such as hallways or a cafeteria, the likelihood of reaching the targeted group is diminished.

When PCTs are embedded in routine care, responsibility for making determinations about informed consent and/or notification is not limited to investigators and review committees but includes the institutions as well. Most obviously, posting general announcements about research requires institutional support. And health care institutions may have requirements for what information can be posted in public areas.

## 9 POTENTIAL BURDENS AND COSTS

Disclosing information to participants can have important value independent of soliciting their consent. It can also have potential negative consequences. For example, if a study is designed to assess the outcomes of standard care, informing individuals that they are participating in a study may alter their behavior and, thereby, decrease the extent to which the results reflect standard care. Individuals who are informed that they are participating in research also may assume that they will be exposed to increased burdens or risks and attempt to opt out or, if that is not an option, seek care elsewhere. Others may have no concerns but seek to opt out anyway. If enough individuals respond in these ways, the generalizability of the study could be undermined, especially if those who opt out tend to be from specific groups.

The costs, like the benefits, of promoting the six goals, vary across studies. Hence, it is not possible to provide specific guidance regarding whether and how to inform individuals. Instead, researchers and review committees should carefully consider the benefits and the costs for each study.

## 10 CONCLUSION

We have argued that providing participants with information about studies conducted with a waiver of research consent can promote six important goals: respect for persons, participant understanding of the research, participant understanding of their contributions, participant ability to voice any concerns, participant engagement, and trust and trustworthiness. Given the ethical importance of these goals, this analysis suggests that informing participants should be the default, with proposals not to communicate any information requiring specific justification.

The importance of these goals, and their relevance, can vary considerably across studies. Hence, rather than endorsing a general approach regarding what information should be provided and how, we have argued that researchers and review committees should tailor the approach to the specific study in question.

Future research will be needed to determine the impact of disclosure. Collaborating with studies that request a waiver of consent could provide the opportunity to conduct embedded studies to assess different types and methods of disclosure, including investigator experiences with notification and participant responses to various modes of notification and different levels of detail provided. In the meantime, when a study is approved with a waiver of research consent, investigators and review committees should consider on a case‐by‐case basis what information, if any, to disclose to participants, and how it will be disclosed.

## FUNDING INFORMATION

This work was supported within the National Institutes of Health (NIH) Pragmatic Trials Collaboratory through cooperative agreement U24AT009676 from the National Center for Complementary and Integrative Health (NCCIH), the National Institute of Allergy and Infectious Diseases (NIAID), the National Cancer Institute (NCI), the National Institute on Aging (NIA), the National Heart, Lung, and Blood Institute (NHLBI), the National Institute of Nursing Research (NINR), the National Institute of Minority Health and Health Disparities (NIMHD), the National Institute of Arthritis and Musculoskeletal and Skin Diseases (NIAMS), the NIH Office of Behavioral and Social Sciences Research (OBSSR), and the NIH Office of Disease Prevention (ODP). This work was also supported by the NIH through the NIH HEAL Initiative under award number U24AT010961. Demonstration Projects within the NIH Pragmatic Trials Collaboratory were supported by the following cooperative agreements with NIH Institutes: ACP PEACE (UG3AG060626, UH3AG060626), EMBED (UG3DA047003, UH3DA047003), ICD‐Pieces (UH2DK104655, UH3DK104655), Nudge (UG3HL144163, UH3HL144163), and TiME (UH2AT007797, UH3DK102384). The content is solely the responsibility of the authors and does not necessarily represent the official views of the NCCIH, NIAID, NCI, NIA, NHLBI, NINR, NIMHD, NIAMS, OBSSR, or ODP, or the NIH or its HEAL Initiative.

## CONFLICT OF INTEREST STATEMENT

L.M. Dember has received compensation from the National Kidney Foundation for her role as Deputy Editor of the *American Journal of Kidney Diseases*, consulting fees from AstraZeneca, Cara Therapeutics, and Merck, and compensation for serving on Data and Safety Monitoring Boards for the National Institute of Diabetes and Digestive and Kidney Diseases and Data Monitoring Committees for CSL Behring and Vertex Pharmaceuticals. PM Ho reports research grants from the Department of Veterans Affairs, NHLBI, and the University of Colorado School of Medicine. He serves as a Deputy Editor for Circulation: Cardiovascular Quality and Outcomes. Dr. Melnick was supported by the National Institute On Drug Abuse of the National Institutes of Health under Award Number UH3DA047003. He also reports receiving grants from the Agency for Healthcare Research and Quality and the American Medical Association. The content is solely the responsibility of the authors and does not necessarily represent the official views of the National Institutes of Health, the Agency for Healthcare Research and Quality, or the American Medical Association. Dr. Angelo Volandes has a financial interest in ACP Decisions, a non‐profit organization developing advance care planning video decision support tools. Dr. Volandes' interests were reviewed and are managed by MGH and Mass General Brigham in accordance with their conflicts of interest policies. David Wendler This work was funded, in part, by NIH intramural funds. However, the opinions expressed are the authors' own. They do not represent the position or policy of the National Institutes of Health. All authors declare that they have no conflicts of interest related to this work.
